# Toward benchmarking theoretical computations of elementary rate constants on catalytic surfaces: formate decomposition on Au and Cu†

**DOI:** 10.1039/d1sc05127j

**Published:** 2021-12-21

**Authors:** Eri Muramoto, Wei Chen, Xiwen Jia, Cynthia M. Friend, Philippe Sautet, Robert J. Madix

**Affiliations:** John A. Paulson School of Engineering and Applied Sciences, Harvard University Cambridge MA 02138 USA rmadix@seas.harvard.edu; Center for Functional Nanomaterials, Brookhaven National Laboratory Upton NY 11973 USA; Department of Chemistry and Chemical Biology, Harvard University Cambridge MA 02138 USA; Department of Chemical and Biomolecular Engineering, University of California, Los Angeles Los Angeles CA 90095 USA sautet@ucla.edu; Department of Chemistry and Biochemistry, University of California, Los Angeles Los Angeles CA 90095 USA

## Abstract

With the emergence of methods for computing rate constants for elementary reaction steps of catalytic reactions, benchmarking their accuracy becomes important. The unimolecular dehydrogenation of adsorbed formate on metal surfaces serves as a prototype for comparing experiment and theory. Previously measured pre-exponential factors for CO_2_ formation from formate on metal surfaces, including Cu(110), are substantially higher than expected from the often used value of *k*_B_*T*/*h*, or ∼6 × 10^12^ s^−1^, suggesting that the entropy of the transition state is higher than that of the adsorbed formate. Herein, the rate constant parameters for formate decomposition on Au(110) and Cu(110) are addressed quantitatively by both experiment and theory and compared. A pre-exponential factor of 2.3 × 10^14^ s^−1^ was obtained experimentally on Au(110). DFT calculations revealed the most stable configuration of formate on both surfaces to be bidentate and the transition states to be less rigidly bound to the surface compared to the reactant state, resulting in a higher entropy of activation and a pre-exponential factor exceeding *k*_B_*T*/*h*. Though reasonable agreement is obtained between experiment and theory for the pre-exponential factors, the activation energies determined experimentally remain consistently higher than those computed by DFT using the GGA–PBE functional. This difference was largely erased when the metaGGA–SCAN functional was applied. This study provides insight into the underlying factors that result in the relatively high pre-exponential factors for unimolecular decomposition on metal surfaces generally, highlights the importance of mobility for the transition state, and offers vital information related to the direct use of DFT to predict rate constants for elementary reaction steps on metal surfaces.

## Abstract 2

The underlying factors that result in the high pre-exponential factors for formate decomposition on Au and Cu(110) surfaces and the origins of differences between experiment and theory that may arise are reported.

## Graphical abstract

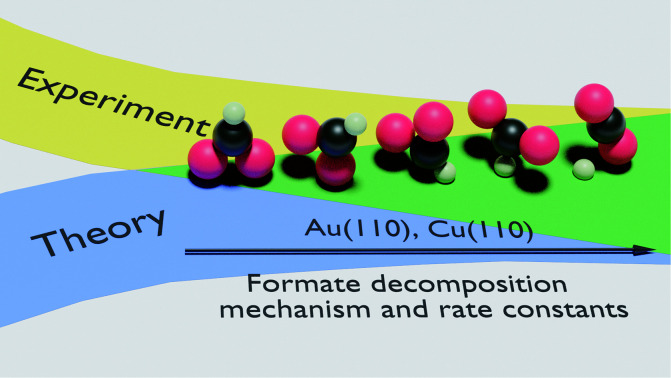

## Introduction

Quantitative prediction of the performance of certain catalytic materials in normal reactor conditions from fundamental mechanistic and kinetic knowledge is becoming a real and exciting possibility. Recently microkinetic modelling and fundamental studies on single crystal surfaces were combined to predict the selectivity and conversion of the esterification of a homologous series of aliphatic alcohols over a nanoporous gold catalyst from determination of the kinetics and mechanism for methanol self-coupling on single crystal gold surfaces – assisted by estimates of some rate constants using theory.^1–3^ The success of such predictions relies on the knowledge of each of the elementary reaction steps comprising the catalytic cycle and accurate values of both the pre-exponential factor and activation energy of each step. With computational advances and the advent of density functional theory, it is conceivable that these rate constants, both their pre-exponential factors and activation energies, could be computed sufficiently accurately to provide these predictions, obviating the need for experiments. However, it is well known that such computations are time-consuming and subject to substantial error, and whether the computations themselves will give quantitatively correct values is unknown. Furthermore, the experimental measurements must themselves be critically evaluated. It seems apparent, that an experimental database of *reliably* measured rate constants is important to which to compare to theory. The well-controlled conditions of ultrahigh vacuum are ideally suited for such determinations using the methods of temperature programmed reaction spectroscopy.^4–9^ In many cases reaction intermediates can be synthesized and isolated and the pertinent rate constants for their reactions measured accurately. In this paper the experimental and theoretically determined rate constants for the decomposition of formate adsorbed on Au(110) and Cu(110) surfaces are compared for this purpose.

In its own right, understanding the thermodynamic and kinetic processes of formate (HCOO) decomposition on catalytic surfaces is important for developing efficient processes of practical importance. Formate is the key intermediate in the catalytic decomposition of formic acid (HCOOH) to CO_2_ and H_2_, which is a promising process for hydrogen storage^10^ and fuel cell applications.^11^ The reverse process, in which CO_2_ is electrochemically transformed into formic acid,^12^ is among the most feasible approaches to commercial reduction of the greenhouse gas. Furthermore, formate is also an intermediate in the oxidative reactions of aldehydes and alcohols,^13,14^ in which formate produced by over-oxidation can act as a poison due to its strong binding to the surface.

Decomposition of formate has been widely investigated on single crystals,^15–20^ including the (110) surfaces.^21–35^ On Cu(110) formic acid undergoes dissociative adsorption to form H_2_ and formate, which decomposes to CO_2_ and H_2_.^21,28^ A well-ordered 2D condensed phase of formate (with a *c*(2 × 2) ordering) has been observed with scanning tunneling microscopy (STM) on Cu(110) resulting from reacting formic acid with 0.25 ML of pre-adsorbed oxygen.^36^ Neither the clean Ag(110) nor Au(110) surface reacts with formic acid, but pre-adsorbed atomic oxygen species reacts with formic acid like a Brønsted base and forms surface formate:^22,31,37^

1 2HCOOH_gas_ + O_ads_ → 2HCOO_ads_ + H_2_O_gas_

The decomposition products of formate on Ag(110) are CO_2_ and H_2_.^37^ The stable configuration is bidentate on both Cu^30^ and Ag,^37^ as evidenced by vibrational spectroscopy. On Au(110) at low initial oxygen coverages, the resulting low formate coverage produces primarily CO_2_ with a small fraction of the hydrogen atoms released reacting with formate to regenerate formic acid.^23,25^ At higher coverages of atomic oxygen, the reaction of formic acid is incomplete due to site blockage, and H_2_O is also formed by the reaction of unreacted atomic oxygen with hydrogen released from the formate species.^31^

The pre-exponential factors for CO_2_ production have been measured for the (110) surfaces of Cu and Ag. They both exceed 6 × 10^12^ s^−1^ – the “universal frequency” of *k*_B_*T*/*h* that arises from harmonic transition state theory (TST). For example, a pre-exponential factor and activation energy of 9.4 × 10^13^ s^−1^ and 133.3 kJ mol^−1^ were obtained for CO_2_ production from formate on Cu(110),^21^ whereas 8 × 10^15±0.5^ s^−1^ and 125 ± 6 kJ mol^−1^ were obtained on Ag(110).^22^ These pre-exponential factors suggest an increase in the entropy of the transition state compared to that of the reactant state for the elementary step of formate decomposition.

Theoretical studies, especially first-principles calculations, have provided a complementary understanding of the reaction mechanisms on an atomic level.^38–41^ Liu *et al.* reported a bidentate configuration of formate on Au(111) and an activation energy of 0.70 eV (68 kJ mol^−1^) for its decomposition.^38^ Yoo *et al.* mapped out the energy diagram for formic acid decomposition on various metal surfaces and established a scaling relationship to estimate adsorption energies of reaction intermediates based on two independent descriptors.^39^ A microkinetic model was developed to predict the varying activity on metals. Sautet *et al.* compared the formate and carboxyl pathway for formic acid decomposition on Pd^41^ and Ni,^40^ and identified the important roles of electrochemical potential on the reaction kinetics.

Nakamura and co-workers have previously published extensive work on formate synthesis and decomposition on copper surfaces.^20,42–45^ Specifically, for Cu(110) they measured activation energies of 145 and 157 kJ mol^−1^ and a pre-exponential factor of 1.22 × 10^16^ s^−1^ for continuous bidentate formate decomposition from 0.25 to zero ML formate coverage. The pre-exponential factor is 10^3^ times that reported in the previous work of Ying *et al.*^21^ In addition, using density functional theory (DFT) and the nudged elastic band (NEB) method, they suggested a monodentate-like transition state (modelled in the zero-coverage limit without frequency analysis), yielding an activation energy of 138.4 kJ mol^−1^. They did not compute preexpontential factors, but, borrowing from Bowker *et al.*,^36^ they suggest that rotation of the O–C–O plane of the bidentate structure toward the surface may be important in determining its value. In our study we used not only the NEB method, but also the dimer method and harmonic frequency analysis, to ensure the convergence of the transition state structures and verify that only one degree of freedom is associated with a negative force constant. In addition, we included the van der Waals corrections. To the best of our knowledge, there exists no study to offer detailed insights into the source of the entropy gain in the transition state that may be related to the high pre-exponential factor observed experimentally.

In this work, the rate constant for formate decomposition on Au(110) was measured to complement the existing information for Cu(110), and the reaction was analyzed by DFT on both surfaces. The activation energy and pre-exponential factor of formate decomposition on Au(110) was determined using the heating rate variation method.^6^ Further, the most stable configurations of adsorbed formate on Au(110) were determined by STM in order to better define the configuration of the formate prior to measuring its decomposition rate constant. The stable and metastable configurations of formate were calculated, allowing identification of the possible decomposition paths. Kinetic parameters were then computed and compared with the experimental results from this and previous work.^21^

## Methods

### Experimental

Temperature-programmed reaction spectroscopy (TPRS) and high-resolution electron energy spectroscopy (HREELS) were performed in an ultra-high vacuum (UHV) chamber with a base pressure below 3 × 10^−10^ torr, described in detail previously.^46^ The Au(110) single crystal (SPL, 8 mm diameter, 2 mm-thick) was radiatively heated using a tungsten filament placed approximately 3 mm behind the crystal. The temperature was monitored by a K-type thermocouple inserted into a pinhole on the side of the crystal. TPRS was performed using a triple filter Hiden quadrupole mass spectrometer (QMS, Hiden HAL/3F). HREELS was conducted using a HREEL spectrometer (LK-2000) with a primary beam energy of 7 eV and 60° specular geometry. Spectra were normalized by the average intensity in the flat background region as described previously.^46^ An established cleaning process^47^ with Ar^+^ and O_3_ was used in the initial cleaning of the crystal. There was no residual carbon species from formate decomposition for all coverages, but the surface was exposed to O_3_ and annealed at 800 K for 5 min between temperature-programmed experiments to ensure that the decomposition reaction was conducted with the same initial surface state.

Scanning tunneling microscopy (STM) experiments were conducted in a separate UHV chamber (Omicron VT-STM) with a base pressure below 1.5 × 10^−10^ torr. A different Au(110) single crystal (Princeton Scientific Corp.) was cleaned with Ar^+^ and O_3_ until the surface was clean when imaged with STM. The crystal was cooled to ∼160 K by contacting the back of the crystal with a cooling block, which was cooled using cold nitrogen gas. A mechanically cut Pt/Ir tip (Veeco Instruments, Inc) was used for scanning. The *in situ* measurements were conducted by dosing formic acid onto the oxygen-covered Au(110) using a directed dosing tube located 2 cm from the crystal while scanning.

Adsorbed atomic oxygen was prepared by exposing the clean surface to O_3_ produced by a commercialized O_3_ generator (Ozone Engineering, LG-7) with a concentration monitor (Teledyne Instruments, model 454H), at 300 K in all experiments. For TPRS and HREELS experiments, the coverage of oxygen adatoms was calibrated by comparing the integral of the O_2_ recombination peak at 560 K to that of the O_2_ recombination peak for mono-layer coverage.^48^ For STM experiments the coverage was calculated by an established procedure^49^ from the ratio of the clean Au area and oxygen covered area measured using image analysis software (Scanning Probe Image Processor, SPIP, Image Metrology). The formate intermediate was prepared by dosing formic acid (Sigma Aldrich, ACS reagent purity) onto oxygen-covered Au(110) at 200–250 K in order to prevent the accumulation of unreacted formic acid and water.^31^ Gases dissolved in formic acid were removed by freeze–pump–thaw cycles and water was removed with anhydrous calcium sulfate (indicating Drierite, W. A. Hammond Drierite Co., Ltd). Formate coverages were determined by assuming that 0.05 ML of atomic oxygen yields 0.1 ML of formate^25^ and that the integral of the CO_2_ desorption peak scales linearly with formate coverage. The coverages determined this way were in good agreement with the coverages that were obtained alternatively by comparing the integral of the CO_2_ to the integral of atomic oxygen peak at saturation, after signal correction to account for fragmentation and the differences in ionization cross section and mass spectrometer transmission and detection coefficients (ESI Fig. S1 and Table S1†).

### Computational details

Density functional theory (DFT) calculations were performed using the Vienna *ab initio* simulation package (VASP),^50^ with the projector-augmented wave (PAW) potentials^51^ and the generalized gradient approximation parameterized by Perdew–Burke–Ernzerhof (GGA–PBE)^52^ for the exchange–correlation functional. MetaGGA–SCAN^53^ were also employed for the activation energy calculations. The energy cutoff was 400 eV for the plane-wave basis sets. The DFT-TS method^54^ was used to account for the van der Waals interactions. The lattice constants of Cu and Au were obtained via energy minimization. The Cu(110) surface was modeled by a slab of four-atomic-layer-thick and a 3 × 4 supercell, while the Au(110) surface by a slab of five-atomic-layer-thick 4 × 4 supercells. Different slab thickness and supercell sizes were used for Cu and Au because Au(110) presents a missing row 1 × 2 reconstruction while Cu(110) remains unreconstructed. 5 × 5 × 1 and 4 × 5 × 1 *k*-point meshes^55^ were used for the Cu and Au supercells, respectively. The vacuum region was thicker than 15 Å to ensure decoupling between neighboring images. During relaxation, the top three layers of metal atoms and all atoms in the adsorbates, were fully relaxed until the force on each atom was smaller in magnitude than 0.002 eV Å^−1^. The climbing image nudged elastic band (CI-NEB) method,^56^ the dimer method,^57^ and harmonic frequency analysis were used to systematically and carefully explore several possible reaction paths. The path with the lowest barrier was reported here. The Hessian matrix was calculated by the finite difference method. Four displacements, with a step size of 0.01 Å, were applied along each Cartesian coordinate. The harmonic frequencies and Cartesian displacement of the normal modes were obtained from diagonalization of the mass-weighted Hessian matrix. The atomic structures and displacement of the normal modes were visualized by VESTA.^58^

## Results and discussion

### Formate decomposition on Au(110)

**Fig. 1:**
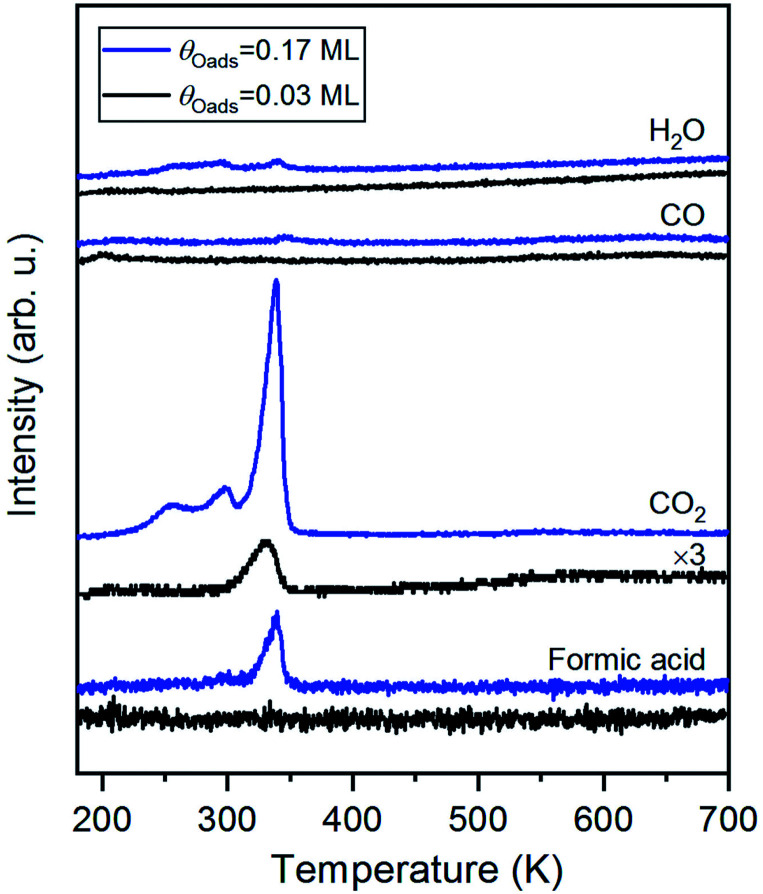
The major product of temperature-programmed reaction of formic acid and oxygen-covered Au(110) is CO_2_; formic acid (HCOOH) and a small amount of H_2_O are produced at higher coverages. 3 MLE of formic acid was dosed onto either 0.03 ML or 0.17 ML O/Au(110) at 200 K, above the desorption temperatures of unreacted molecular formic acid and H_2_O formed by the reaction between adsorbed oxygen species and acidic H of formic acid. Formic acid was also regenerated from the reaction between formate and hydrogen atoms released in formate decomposition. Heating rate was 1 K s^−1^.

Carbon dioxide was the only product detected during temperature-programmed reaction of formic acid on oxygen-covered Au(110) at an initial oxygen coverage of 0.03 ML, which is expected to yield 0.06 ML of formate (Fig. 1 and eqn (1)). At a higher initial oxygen coverage of 0.17 ML, formic acid and a small amount of H_2_O were produced, in agreement with prior studies performed on gold single crystals,^25,31,59^ and two low-temperature shoulders developed in the CO_2_ peak. The evolution of these products is reaction-limited, as molecular CO_2_,^60^ formic acid and H_2_O themselves desorb below 200 K on Au(110) (ESI Fig. S2†). CO and H_2_ were not detected. All adsorbed oxygen was consumed by formic acid based on the absence of the O_2_ recombination peak at 550 K. The TPRS results thus indicate the formation of formate by reaction of formic acid with preadsorbed O, as concluded in earlier work.^31^ More exact quantification of the ratio of CO_2_ and HCOOH requires knowledge of the angular flux distribution of these products, which could differ significantly,^44,61,62^ and is not available in the literature.

### HREELS of formate on Au(110)

**Fig. 2:**
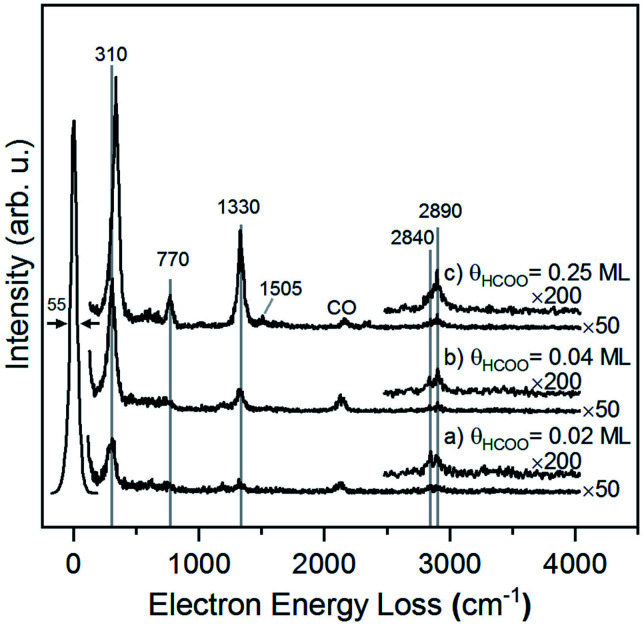
Formate forms a bidentate structure after reaction of formic acid with adsorbed oxygen on Au(110) based on interpretation of high resolution electron energy loss spectra. Data shown were obtained after preparing (a) 0.02 ML, (b) 0.04 ML and (c) 0.25 ML of formate by exposing an oxygen-covered Au(110) to 3 MLE of formic acid at 250 K. All spectra were collected after cooling to 110 K. Peak assignments are given in Table 1.

The structure of formate on metal surfaces has been extensively studied; the highly symmetric bidentate configuration identified previously on Cu and Ag surfaces was also observed on Au(110) in this study. Formate on Au(110) exhibits a bidentate configuration in the *C*_2v_ point group, as evidenced by high resolution electron energy loss (HREEL) spectra for formate coverages of 0.02, 0.04 and 0.25 ML (Fig. 2). The vibrational modes were assigned by comparison with DFT calculations for Au(110) and previously reported HREEL spectra of bidentate formate on Cu(110),^36^ Ag(110)^37^ and Cu(100)^63^ (Table 1). The three intense bands observed for formate on Au(110) were assigned to the Au–oxygen stretch (310 cm^−1^), OCO bend (770 cm^−1^), and OCO symmetric stretch (1330 cm^−1^), denoted as ν(Au–O), δ(OCO), and ν_s_(COO), respectively. Broad, less intense bands at 2830 cm^−1^ and 2890 cm^−1^ were assigned to the CH stretch, denoted as ν(CH). The out-of-plane CH bend at 1010 cm^−1^ and OCO anti-symmetric stretch at 1505 cm^−1^ were barely distinguishable from the background at the highest coverage. The observed vibrational frequencies are in excellent agreement with the DFT calculations for bidentate formate reported below. Moreover, these assignments are characteristic of bidentate formate that belongs to the *C*_2v_ point group with the *C*_2_ axis perpendicular to the surface.^29^ Formate on Cu(110), Ag(110) and Cu(100) after annealing to 300 K,^36^ 300 K ^37^ and 400 K,^63^ respectively, all gave very similar vibrational spectra. The absence of a mode near 1700 cm^−1^ rules out the monodentate configuration.

**Table 1:** Vibrational frequencies of formate adsorbed on coinage metal surfaces (cm^−1^)

Au(110)^a^				Cu(110)^36^		Ag(110)^37^	Cu(100)^63^
Vibrational mode	Experiment	DFT bidentate	DFT monodentate	Experiment bidentate	Experiment monodentate	Experiment	Experiment
ν(Au–O)	310–335					280	340
δ(O–C–O)	770			767		770	760
δ(O–C=O)					787		
π(CH)	Weak					1050	Weak
ν(C–O)			1158		1266		
ν_s_(COO)	1330	1293		1355		1340	1330
δ(CH)	Obscured				1381	Obscured	Obscured
ν(C=O)			1714		1640–1670		
ν_a_(COO)	1505	1527			2865	1640	1640
ν(CH)	2840, 2890	2929	2083	2849	2945	2900	2840, 2890
ν_a_(COO) + δ(CH)				2930	2930		

a This work.

The presence of lateral interactions between formate species is indicated by the small shift in the ν(Au–O) mode with increasing coverage. The ν(Au–O) mode shifts from 310 cm^−1^ to 335 cm^−1^, while other vibrational modes remain unaffected. This slight increase in frequency suggests that the bond anchoring formate to the surface becomes stronger and that formate is stabilized with increasing coverage.

### CO_2_ production from formate

**Fig. 3:**
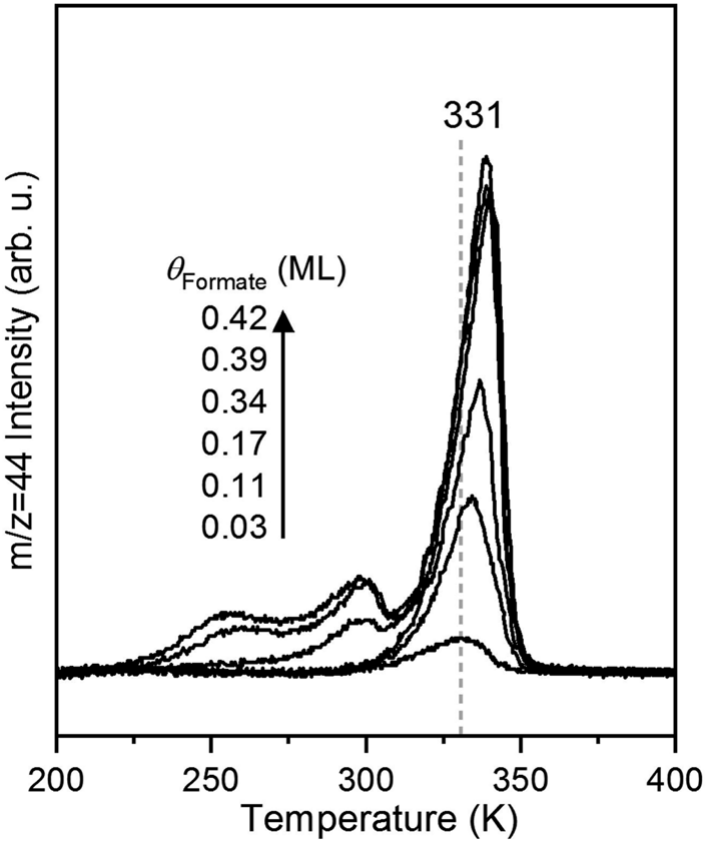
The kinetics of CO_2_ formation from formate decomposition depends on the initial coverage of oxygen on the Au(110) surface based on shifts in the peak temperature and development of multiple peaks. The formate coverages are 0.03, 0.11, 0.17, 0.34, 0.39 and 0.42 ML. Formic acid was dosed onto the oxygen-covered surface at 200 K followed by a linear heating at 1 K s^−1^.

The rate of CO_2_ production depends on the surface coverage of formate (Fig. 3). At the lowest coverage examined by TPRS, CO_2_ evolved in a single peak with a peak temperature at 331 K. With increasing initial formate coverage (0.03, 0.11, 0.17, 0.34, 0.39 and 0.42 ML), the amount of formate near 331 K increased, and a second peak was observed at 300 K at 0.34 ML followed by a third peak at 250 K at 0.39 ML and higher. There is a clear shift in the peak at 331 K to 339 K with increasing coverage, consistent with attractive interactions among formate species^4,6^ in this bidentate configuration. The appearance of the second and third desorption peaks suggests the filling of multiple formate binding configurations which produce higher decomposition rates at higher coverages, perhaps resulting from repulsive interactions at these higher coverages. In this paper the kinetic analysis is confined to the low coverage regime.

### Measurement of rate constant parameters

The decomposition of the formate intermediate on Au(110) follows simple first-order kinetics at low initial coverages, based on analysis of the CO_2_ temperature-programmed reaction peak. The rate of the surface reaction is described by the Polanyi–Wigner equation:^5^2
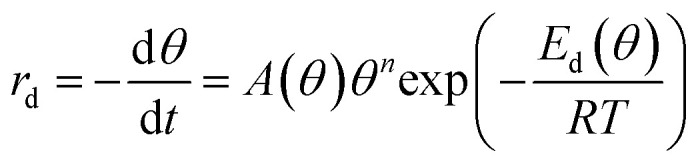
where *θ* is the adsorbate coverage, *A* the pre-exponential factor, *n* the reaction order, and *E*_d_ the activation energy. At the lowest initial formate coverage the peak symmetry for evolution of CO_2_ (the ratio of the integral under the curve above peak temperature, *T*_p_, to the total integral of the peak) is in excellent agreement with the value expected theoretically for a first order reaction (0.37) (ESI Fig. S3†). Similarly an isothermal analysis^21^ of the rate *versus* coverage yields a reaction order of unity at low coverage (ESI Fig. S3†). Both methods demonstrate that the evolution of CO_2_ from formate on Au(110) can be described by first-order kinetics at low coverages.

**Fig. 4:**
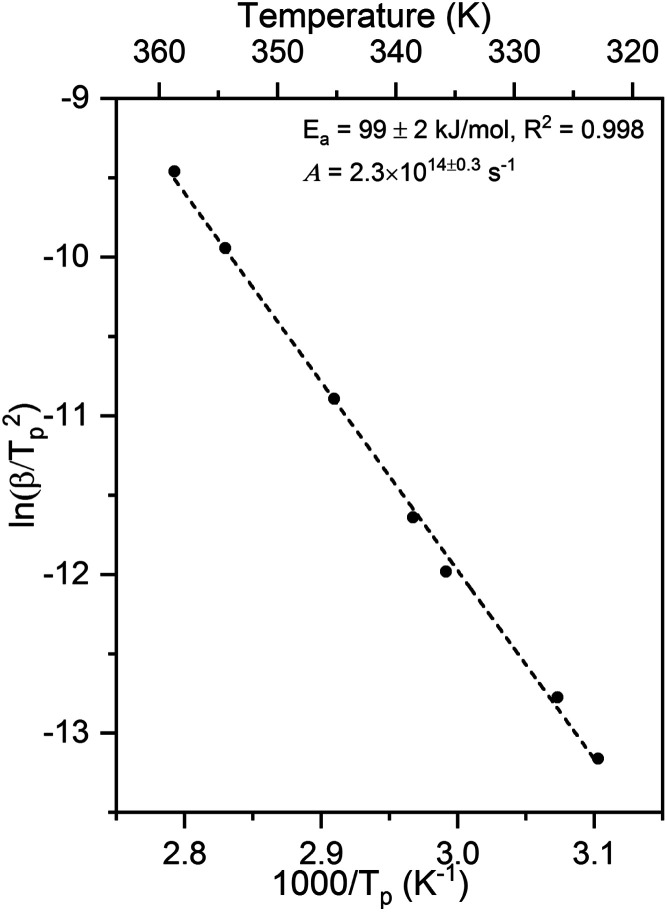
Activation energy of 99 ± 2 kJ mol^−1^ and pre-exponential factor of 2.3 × 10^14±0.3^ s^−1^ were obtained for 0.04 ML of formate decomposition on Au(110) by heating rate variation analysis.^6^ Heating rates of 0.2, 0.3, 0.7, 1, 2.2, 6, and 10 K s^−1^ were used. Formic acid was dosed onto 0.02 ML O/Au(110) at 250 K. An *E*_a_ of 99 ± 2 kJ mol^−1^ was obtained from the slope by least squares fitting and an *A* of 2.3 × 10^14±0.3^ s^−1^ was obtained by substituting *E*_a_ and pairs of *β* and *T*_p_ into eqn (3).

The activation energy and pre-exponential factor for formate decomposition at 0.04 ML were determined to be 99 ± 2 kJ mol^−1^ and 2.3 × 10^14±0.3^ s^−1^, respectively, by analysis of the peak temperature shift as a function of heating rate (Fig. 4) according to the relationship^6^3
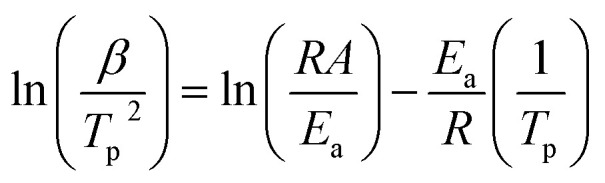


The activation energy measured corresponds to that of formate decomposition because the evolution of CO_2_ is reaction-limited. The TPR spectra simulated using these experimentally determined *E*_a_ and *A* are in excellent agreement with the measured spectra, further confirming their accuracy (ESI Fig. S4†). That *A* is an order of magnitude higher than *k*_B_*T*/*h*, or 2.3 × 10^13^ s^−1^ at 350 K, indicates that the transition state for CO_2_ formation from formate on this coinage metal (110) surface has a higher partition function than the reactant state – the adsorbed formate.

**Fig. 5:**
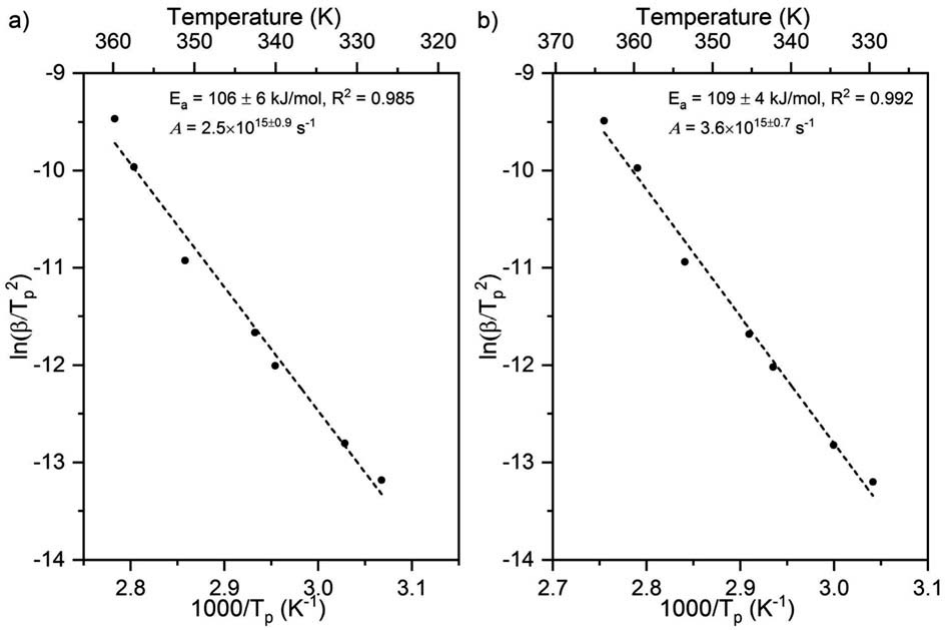
Activation energy and pre-exponential factor of formate decomposition on Au(110) at formate coverages of (a) 0.10 ML and (b) 0.25 ML were determined by heating rate variation analysis.^6^ The activation energy was 106 ± 6 kJ mol^−1^ and pre-exponential factor was 2.5 × 10^15±0.9^ s^−1^ for 0.10 ML. The standard deviation for this coverage was higher than those for the other coverages. For 0.25 ML the activation energy was 109 ± 4 kJ mol^−1^ and pre-exponential factor was 3.6 × 10^15±0.7^ s^−1^. Formic acid was dosed onto oxygen-covered Au(110) at 250 K and heating rates of 0.2, 0.3, 0.7, 1, 2.2, 6, and 10 K s^−1^ were used.

Larger activation energies and pre-exponential factors were obtained for formate decomposition at formate coverages of 0.10 and 0.25 ML (Fig. 5 and Table 2). There is a clear compensation effect^64^ for this simple reaction over this range of surface coverage. It is important to note that the standard deviation of both kinetic parameters for 0.10 ML are higher than the values for 0.04 ML and 0.25 ML. Although there were secondary and tertiary peaks for the highest coverage, peak fitting of the spectra confirmed that they do not affect the *T*_p_ of the primary peak. The combinations of rate constant parameters are in agreement with other methods^9^ (ESI Fig. S5 and S6†). These measurements suggest that the activation energy and pre-exponential factor increase slightly with increasing coverage. Values of the pre-exponential factor exceeding 10^15^ s^−1^ were observed at these higher coverages. The rate constant parameters at the higher coverages were not addressed by DFT in this paper.

**Table 2:** Experimentally determined activation energy *E*_a_ and pre-exponential factor *A* of formate decomposition on Au(110) and Cu(110)

	Au(110)^a^			Cu(110)^21^
	0.04 ML	0.10 ML	0.25 ML	b
*E*_a_ (kJ mol^−1^)	99 ± 2	106 ± 6	109 ± 4	133
*A* (s^−1^)	2.3 × 10^14±0.3^	2.5 × 10^15±0.9^	3.6 × 10^15±0.7^	9.4 × 10^13^

a This work.

b Negligible coverage dependence.^21^

### STM of formate on Au(110)

**Fig. 6:**
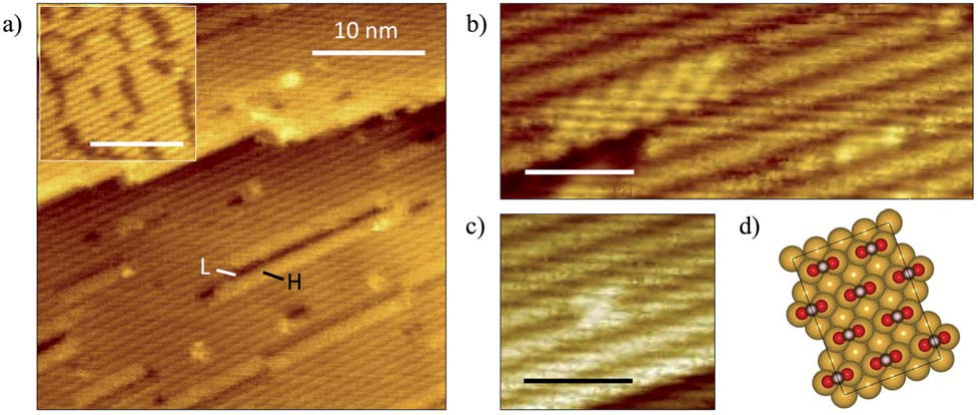
Exposure of oxygen-covered Au(110) surface to formic acid results in formation of formate clusters exhibiting a *c*(2 × 2) structure and deconstruction of the underlying Au-(1 × 2) surface. (a) STM image showing formate condensation with inset showing the initial oxygen-covered surface at 0.08 ML. Surface was exposed to formic acid at 200 K. The surface lifetime of water and formic acid are less than a few seconds at this temperature, and only that of formate is significantly long for it to be imaged in these STM experiments; scale bar in inset: 10 nm, image size: 35 × 35 nm^2^, sample bias: +1.5 V, tunneling current: 0.1 nA. The bright linear features (high-domains) are formate chains while dark linear features are lower domains of vacant neighboring Au rows descendent from the (1 × 2) reconstruction which were displaced.^65^ (b) High-resolution STM image shows formate clusters along the close-packed rows and formate strings on unperturbed Au-(1 × 2). Image size: 4 × 10 nm^2^. (c) A single unit cell of *c*(2 × 2) structure. Image size: 4 × 4 nm^2^. Scale bar in (b and c): 2 nm. (d) Schematics illustrating the *c*(2 × 2) formate structure on a deconstructed (1 × 1) Au surface.

The state of aggregation of formate on Au(110) was examined by scanning tunneling microscopy. Oxygen atoms were first adsorbed using an ozone source previously described.^49^ Attempts to stabilize formate with initial oxygen coverages below 0.06 ML in these STM experiments were unsuccessful, so oxygen coverages of 0.06–0.17 ML were employed. The STM images collected at this coverage range showed oxygen in condensed island structures (Fig. 6a, inset), in agreement with previous reports.^49^ Exposure of the surface to formic acid at 200 K led to the formation of small islands of formate in a *c*(2 × 2) structure (Fig. 6a–c) similar to that observed for acetate on Au(110) in earlier work.^65^ The co-existence of high (H) domains, low (L) domains and undisturbed Au-(1 × 2) areas was observed across the terraces. The self-assembly of formate induce migration of Au atoms from neighboring rows in the (1 × 2) structure, resulting in formate adsorbed on an underlying Au-(1 × 1) surface. The absence of formate structures at the lower oxygen coverages is strongly suggestive of the random adsorption of formate below coverages of 0.10 ML, though this matter is not entirely clear.

A variety of formate aggregates, all directed along the close-packed rows, was observed, ranging from 2D chains a few-rows-wide to short strings on a single row (Fig. 6b and c). Mixed lengths of low domains (dark) were also discernible, and they appeared to correlate with the length of neighboring formate clusters. The formate chains and clusters exhibit a *c*(2 × 2) molecular arrangement (Fig. 6d), which is consistent with low-energy electron diffraction patterns obtained previously for a global coverage of ∼0.25 ML of formate on Au(110).^25^ The structure of the surface unpopulated by formate remained undisturbed.

### Theoretical computation of the activation energy and pre-exponential factor for formate decomposition. Determination of the transition state and its effect on the partition function

**Fig. 7:**
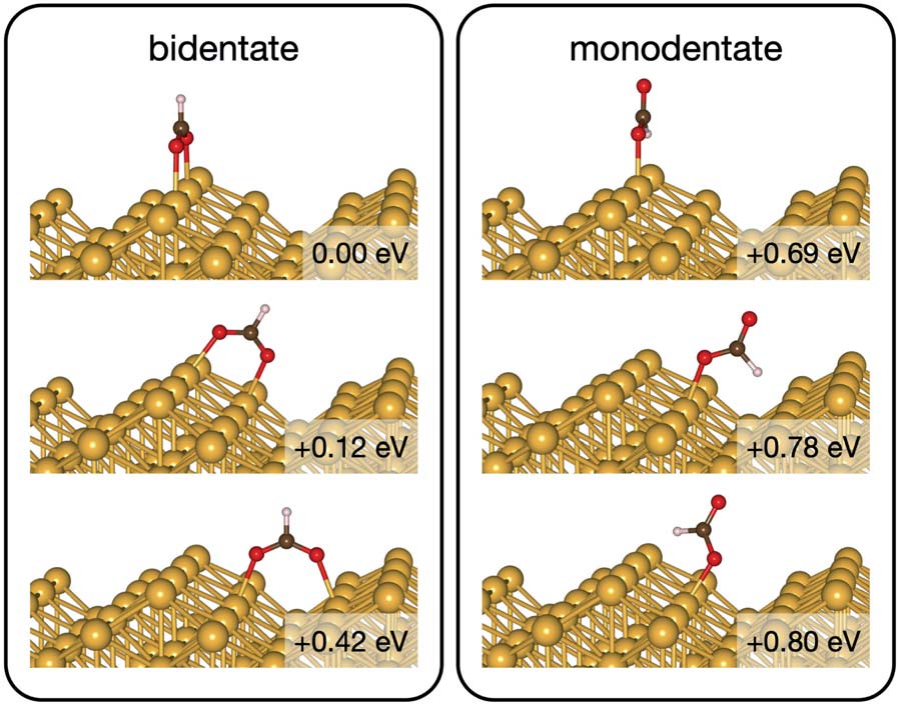
The intermediate configurations of formate on Au(110)-1 × 2 categorized into two groups – bidentate and monodentate. The labelled relative energies are with respect to the most stable configuration. A more positive relative energy means a less stable structure.

**Fig. 8:**
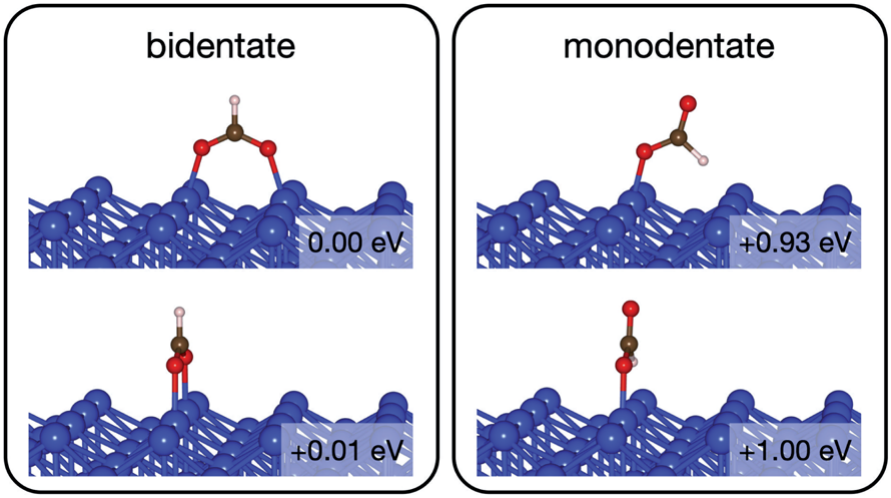
The intermediate configurations of formate on Cu(110).

Initially analysis was performed using GGA–PBE. Computations indicate that formate prefers a bidentate adsorption configuration on both Au(110) (Fig. 7) and Cu(110) (Fig. 8) surfaces. On the most stable configuration of the Au(110) surface (the missing row reconstructed Au(110)-1 × 2 surface, Fig. 7), which is the configuration adopted by the pre-adsorbed oxygen atoms, each of the two oxygen atoms in formate binds with one of two neighboring Au atoms along the row, and the C–H bond points upright. The alternative bidentate configurations are higher in energy by 0.12 and 0.42 eV, respectively (Fig. 7). To break the C–H bond in formate, the formate has to first rotate into a monodentate configuration where the C–H bond is tilted towards the surface. It is therefore important to investigate the structures and energies of these monodentate configurations. Their energies were found to be higher than the most stable bidentate configuration by at least 0.69 eV (Fig. 7). In the most stable monodentate structure, the molecule forms one O–Au bond with the surface and all the atoms in formate remain in a plane above the Au row.

On the Cu(110) surface the missing row reconstruction is not favored and two of the bidentate structures are almost degenerate in energy. In one structure formate lies between two Cu atoms that are from two neighboring parallel rows. In the other structure, formate adopts the same short-site bridging configuration as that in the most stable structure on Au(110)-1 × 2. These two structures are expected to have similar population on the Cu surface although only the short-bridge was generally considered to be the experimentally measured.^66,67^ The lowest energy monodentate structure on Cu, where one O is at the tilted atop site and the C–H bond points towards the Cu atom in the neighboring row, is different from that on Au. Such difference in the stability hierarchy of various monodentate structures is probably due to the reconstructed *versus* unreconstructed nature of the two surfaces.

**Fig. 9:**
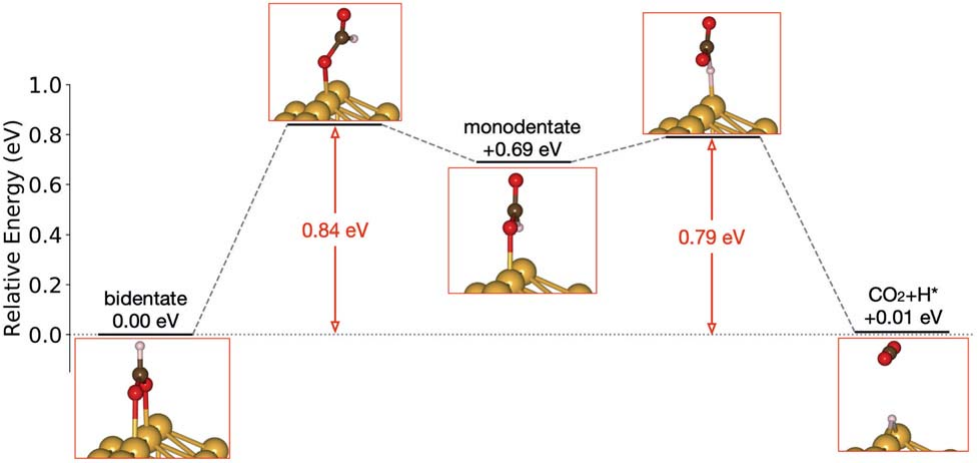
Reaction pathway of formate decomposition on Au(110)-1 × 2. The relative energies were calculated from internal energies.

**Fig. 10:**
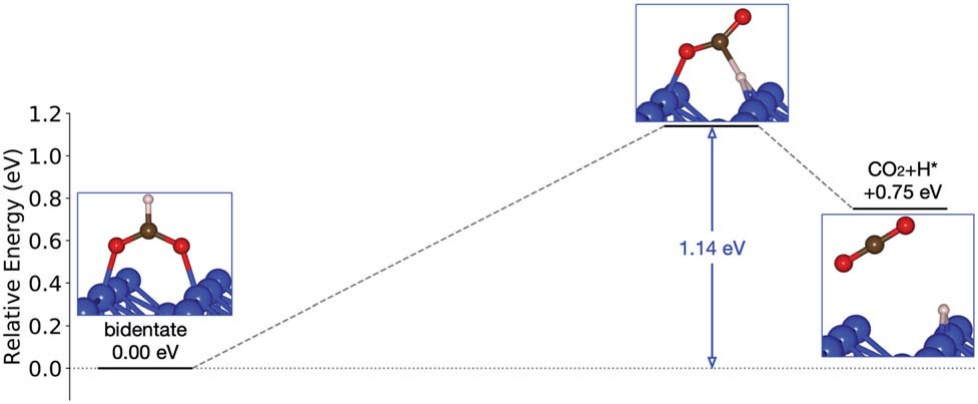
Reaction pathway of formate decomposition on Cu(110). The relative energies were calculated from internal energies.

Starting from the most stable structure(s), the potential energy paths of formate decomposition were identified. On each surface several possible paths were compared to determine the most probable one (Fig. 9 and 10). Important differences were discovered between the energetics features on Au and on Cu. From a thermodynamic point of view, formate decomposition to CO_2_ plus atomic H is almost thermoneutral on Au(110), whereas on Cu(110) this reaction is highly endothermic by 0.75 eV. This difference is primarily due to the stronger O–metal bond on Cu(110).

Accordingly, the kinetic analysis also shows that the reaction on Au is more accessible than on Cu. Two successive kinetic barriers have to be overcome on Au(110). The first one, rotation from bidentate to monodentate configuration, bears a barrier of 0.84 eV. The second one, C–H bond breaking of monodentate formate, requires a small activation energy of 0.10 eV, which is less than the activation energy for reconversion to the bidentate state. The transition state in the first step is higher in energy than in the second step, suggesting that once formate rotates to a monodentate configurations, it tends to break the C–H bond rather than to rotate back to bidentate. Therefore, rotation from bidentate to monodentate is the rate-limiting step. An interesting observation is that, despite formate being upright above the Au row in both bidentate and monodentate configurations, formate must tilt from the upright plane in the transition state between them. When the molecule is constrained to lie within the plane, the calculated transition state showed more than one imaginary frequency in the harmonic frequency analysis.

On the Cu(110) surface there is only one transition state, with an activation energy of 1.14 eV, and the rate-limiting step involves both the bidentate-to-monodentate rotation and the C–H bond breaking rather than the bidentate-to-monodentate rotation. During the structural evolution from bidentate to monodentate and further to the transition state, the adsorption site where O binds with Cu changes from one-fold to two-fold and then back to one-fold site. Because of the dissociating H, the O–C–O angle in the transition state (147°) is much larger than that in bidentate (130°) and in monodentate (125°). This angular information suggests a late transition state whose electronic structure should be more similar to that of the final state.

On both Au and Cu, the transition state of formate interacts less rigidly with the surface compared to the initial binding state of the reactant, where two rigid metal–O bonds exist. We therefore expected the transition state to gain entropy in the transition state from such increased freedom of motion. To quantify this effect harmonic frequency analysis was performed, and the pre-exponential factors were estimated with the known structures of the transition states.

A wide range of harmonic frequencies for the normal modes of both reactant and transition states was seen in the frequency analysis (ESI Fig. S7 and S8†). The construction of the Hessian matrix included the coordinates of the adsorbate atoms and of 2(6) Au(Cu) atoms in the top layer that are near the adsorbate. The choice of more Cu atoms in the Hessian matrix is because the reaction on Cu involves metals atoms from two rows, while on Au it involves only one. The low frequency range of the spectrum (frequencies smaller than about 100 cm^−1^) overlaps largely on both Au and Cu for both the reactant and transition states, which suggests that the vibrational partition functions for the reactant (*Q*^RS^) and transition states (*Q*^TS^) would be *similar*.

Using the same equations reported previously,^68^ the harmonic pre-exponential factor was estimated from the frequencies (Table 3). At each temperature the pre-factors on the two surfaces are similar, both being roughly three times higher than *k*_B_*T*/*h.* This observation is qualitatively consistent with the experimental result on Cu(110)^21^ and suggests a significantly increased entropy in the transition state compared to the reactant state. Quantitatively, the theoretical pre-factor on Cu(110) of 2.9 × 10^13^ s^−1^ at 460 K (the peak temperature of CO_2_ evolution^21^) is still below the experimental value of 9.4 × 10^13^ s^−1^, though the difference is significantly less than an order of magnitude. On Au(110) the theoretical pre-factor is 2.3 × 10^13^ s^−1^ at 350 K (the peak temperature of CO_2_ evolution measured in this work), showing a larger difference with the experimental value of 2.3 × 10^14±0.3^ s^−1^. The deviation from *k*_B_*T*/*h* is determined largely by the change in frequency of a single mode, attributed to the floppy motion of the formate. A more comprehensive discussion of kinetic parameters between experiment and theory is given below.

**Table 3:** Theoretically estimated harmonic pre-exponential factor *A* on Au(110) and Cu(110), in comparison to *k*_B_*T*/*h* at 300, 400, and 500 K, respectively. The units of *A* are s^−1^

*T*	*k*_B_ *T*/*h*	*A* = (*k*_B_*T*/*h*) × (*Q*^TS^/*Q*^RS^)	
		Au(110)	Cu(110)
300 K	6.3 × 10^12^	2.0 × 10^13^	1.7 × 10^13^
400 K	8.3 × 10^12^	2.5 × 10^13^	2.5 × 10^13^
500 K	1.0 × 10^13^	2.9 × 10^13^	3.2 × 10^13^

The higher entropy in the transition state is due to the floppier nature of the transition state structure compared to the reactant state. Since the pre-factor is dominantly determined by the low frequency vibrational modes, we zoomed into the frequency spectra in the low-frequency region (ESI Fig. S7 and S8†). On both Au(110) and Cu(110), the three lowest frequency modes in the reactant state all have lower frequencies in the transition state. We visualized the atomic displacements (ESI Fig. S9†) in the lowest frequency modes of the reactant (13 cm^−1^) and transition (6 cm^−1^) states on Au(110). Both modes look like collective hindered rotation around the Au row (floppy motion). The latter mode is softer because formate is less rigidly bound to the surface at the transition state. A Zip file is provided in the ESI† that contains the displacements for all the normal modes on both Au and Cu surfaces.

It has been reported that the pre-exponential factor for dissociation of adsorbed molecules is generally below *k*_B_*T*/*h* and within 1/10 to 1/10^5^ of the pre-exponential factors for desorption of the same molecule at the same temperature.^69^ This is expected for adsorbates that have a relatively free motion in the reactant state that converts to a more strongly constrained transition state, resulting in the decrease in entropy. However, as we have shown for Au(110) and Cu(110) and as have been shown previously for other surfaces,^70^ the pre-exponential factor of formate decomposition generally does not follow this trend. The DFT calculations herein show that formate decomposition is an exception because the initial state of formate is strongly bound to the surface with two oxygen atoms while the activated complex is bound in a floppy monodentate configuration. As such, our results may suggest that a pre-exponential factor exceeding *k*_B_*T*/*h* may be expected for dissociation of other adsorbates where a significant change in the binding configuration (such as a rotation) from a stable initial state to a less stable transition state is necessary for the dissociating atom to interact with the surface atoms.

### Comparing experiment and theory

The pre-exponential factors of formate decomposition determined experimentally on the coinage metal (110) surfaces are greater than *k*_B_*T*/*h*, suggesting that the transition state has a higher entropy than the reactant state. In addition to the value of 2.3 × 10^14^ s^−1^ determined here for Au(110), pre-exponential values of 9.4 × 10^13^ s^−1^ and 8 × 10^15^ s^−1^ were obtained previously for Cu(110)^21^ and Ag(110)^22^ surfaces in other studies, respectively. The mechanistic understanding of the reaction pathway that underlie the pre-exponential factors was provided by DFT calculations, which identified a bidentate initial state and monodentate transition states. These computations, however, result in pre-exponential factors and activation energies lower than the measured values.

The general trends in the pre-exponential factor and activation energies computed for formate decomposition on Cu(110) and Au(110) are overall in agreement with experiment. The initial adsorption configuration of formate identified by DFT on both surfaces is bidentate, which agrees with the mode assignments of the vibrational spectra. The rate-limiting step of decomposition is the rotation from bidentate to monodentate for Au(110) and C–H bond breaking of the monodentate configuration for Cu(110), which, using GGA–PBE, gives zero-point corrected activation energies of 0.80 eV and 0.95 eV, respectively, which are substantially lower than the experimental values of 1.03 and 1.4, respectively (Table 4). It is most appropriate to compare the theoretical values with the experimental values obtained at the lowest coverage studied because at lower coverages the density of formate on the surface appears to most closely resemble the theoretical model, in which a large supercell was used and no van der Waals interactions with other formate species were included. The monodentate transition states result in hc pre-exponential factors of 2.3 × 10^13^ s^−1^ and 2.9 × 10^13^ s^−1^ for Au(110) and Cu(110), respectively, which are somewhat higher than *k*_B_*T*/*h*. These theoretical estimates of activation energy and pre-exponential factor are qualitatively in good agreement with the experimental results for low formate coverages (Table 4), although the DFT estimates are generally lower. On the other hand, the computed rate constants correctly predict the much higher rate of decomposition on Au, compared to Cu(110).

**Table 4:** Pre-exponential factor *A* and activation energy *E*_a_ (corrected for zero point energies) of formate decomposition on Au(110) (*θ*_formate_ = 0.04 ML) and Cu(110): Measurements by heating rate variation analysis and calculations by DFT. The DFT pre-factor and expected *T*_p_ results were estimated from the GGA–PBE results. Two DFS functionals, GGA–PBE and metaGGA–SCAN, were used to calculate the activation energies. The latter functional shows better agreement with the experimental results. Peak temperature *T*_p_ expected for a temperature-programmed reaction spectrum at 5 K s^−1^ heating rate for the obtained rate constants was calculated using eqn (3)

		Au(110)	Cu(110)
*A* (s^−1^)	Experiment	2.3 × 10^14±0.3^	9.4 × 10^13^^a^
	DFT	2.3 × 10^13^	2.9 × 10^13^
*E*_a_ (eV)	Experiment	1.03 ± 0.02	1.4^a^
	DFT	0.80 (GGA–PBE) 1.00 (metaGGA–SCAN)	0.95 (GGA–PBE) 1.15 (metaGGA–SCAN)
Expected *T*_p_ (K)	Experiment	354	489
	DFT	296 (GGA–PBE) 367 (metaGGA–SCAN)	347 (GGA–PBE) 418 (metaGGA–SCAN)

a Ref. 21.

However, due to the considerable underestimation of the activation energies by the GGA–PBE functional, the energetics of the reaction pathway were examined using the SCAN meta-GGA functional, which promises to give more accurate energies.^53,71^ The transition state structure remained almost the same (Fig. S10†). The SCAN functional results in stronger binding of the formate reactant relative to the transition state on both surfaces. Consequently, the activation energy increased by 0.2 eV on both surfaces, bringing the theoretical estimates much closer to the experimental values (see *E*_a_ in Table 4). These results suggest that the SCAN functional may be more suitable for describing the adsorbate/metal systems.

We performed a sensitivity analysis of the pre-factor with respect to the number of atoms in the Hessian calculations and to the functional. We examined the Au(110) surface where the discrepancy between experimental and theoretical pre-factor is bigger. First, varying the number of metal atoms in the Hessian calculations causes a very small change (<7 cm^−1^) to the lowest frequency modes. Therefore, it has minimal effect on the pre-factor. Second, the SCAN functional systematically increases the frequencies in both the reactant and transition states, hence resulting in no change for the pre-factor.

The activation energy difference between Cu(110) and Au(110) is much smaller in theory (0.15 eV) than in experiment (0.37 eV). The 0.22 eV discrepancy can be caused by several reasons: (1) the varying DFT errors on the Cu and Au surfaces; (2) in theory we consider isolated molecules while in experiment molecules condense on both surfaces. The condensation effect on the activation energy can vary on the two surfaces; (3) the experimental values of Cu and Au are from two separate studies, each with their own inherent source of error.

On each surface there are also clear differences in the values between experiment and theory for both the pre-exponential factors and activation energies. Even with the SCAN functional the discrepancy between theory and experiment in *E*_a_ is still 0.25 eV on the Cu surface. A potential explanation for these differences, aside from the inherent accuracy of the functional employed is the stabilization of the reactant state due to attractive lateral interactions among coadsorbed formates, because the computations assumed isolated, single formate species. Prior studies demonstrated that adsorbed atomic oxygen forms condensed structures on Au(110), linking multiple oxygen atoms on neighboring Au atoms along the close-packed rows. At coverages as low as 0.02 ML, they form mostly dimers.^49^ Though no condensed formate structure was observed by STM below 0.10 ML, a variety of aggregated states of low *local* coverage were observed at the higher global coverages. The images shown in Fig. 6a–c reveal aggregates ranging from several contiguous unit cells to a single *c*(2 × 2) unit cell. While a high fraction of formic acid forms dimers in the gas phase, the size of the clusters observed here is larger than what would be expected as a result of formic acid dimer adsorption, which is convincing of lateral interactions between formate.^72,73^ While no direct evidence for stabilization of the formate below 0.10 ML was observed for Au(110), the opposite has been reported for Cu(110).^74–78^ Thus, at low formate coverages, there may be significant attractive interactions among the adsorbed formate species on Cu(110) which contribute to the difference between experiment and theory observed here. Indeed, the inter-molecular van der Waals interaction in the c(2 × 2) condensed network was estimated to be 0.05 eV per formate by DFT (GGA–PBE), which is sufficient to promote more rigid binding of bidentate formate to the surface, stabilizing the reactant state.

As noted above, on Au(110) there is a continuous shift in the lateral interaction stabilization of the formate with increasing coverage, indicating the lateral attractions decrease as the formate coverage becomes low. On Cu(110), however, these degree of stabilization remain unchanged as the formate coverage increases. The most obvious explanation of this difference is the effects of lateral interactions on Cu(110) produce clustering of formate even at low coverages, consistent with previous STM studies.^74–78^

Another possible explanation is the co-existence of multiple kinetic pathways. In the high-dimensional potential energy landscape, in addition to the kinetic pathway that we explored by DFT, multiple reaction paths with slightly higher activation energies may coexist and contribute to the overall reaction rate. In other words, on the microscopic level, the landscape may be heterogeneous. The experiment would measure the effective activation energies and pre-exponential factors resulting from the multiple paths, and these effective quantities should be larger than those of the minimum energy path. It is currently intractable for DFT to sample all these paths, suggesting that a combination of molecular dynamics^79^ and DFT should be explored.

Similar computations were previously conducted for C–H bond breaking of adsorbed C_1_–C_4_ alkoxy species on Cu(110).^68,80,81^ The computed pre-exponential factor for decomposition of methoxy to formaldehyde was very close the experimentally measured value, but, similar to the results reported here with GGA–PBE, the computed activation energy was lower than experiment. The overall trends in reactivity calculated from theory agreed semi-quantitatively with experiment, but the change in pre-exponential factor across the series was minimal. Strikingly, it is known from STM that methoxy also causes reconstruction of Cu(110): *i.e.*, there is an inherent stabilization of methoxy due to aggregation. One must therefore consider that the normal methods of measuring elementary rate constants from TPRS may lead to initial reactant states that are not duplicated in the theoretical computations. Alternative methods, such as molecular beam modulation,^82,83^ should be explored so that rate constants can be determined at higher surface temperatures and lower steady state coverages of the intermediates.

## Conclusions

The experimentally determined pre-exponential factors for CO_2_ formation from formate decomposition on coinage metal (110) surfaces are considerably higher than what is expected from *k*_B_*T*/*h*. DFT calculations performed in this work demonstrate that this originates from the monodentate transition states that are less rigidly bound than the initial, bidentate configurations, which result in higher entropy of activation and thus higher pre-exponential factor. This study provides insight into the underlying factors that result in the relatively high pre-exponential factors for formate decomposition on metal surfaces. The study also reveals that there may be fundamental factors in both experiment and theory that lead to differences in the values of rate constant parameters determined from the two approaches. Suggestions are given for obtaining more accurate values of the rate constants from both experiment and theory.

## Data availability

All the data that support the findings of this study are available within the paper and its ESI† or from the corresponding author upon reasonable request.

## Author contributions

E. M. performed TPRS, HREELS and STM. W. C. performed theoretical calculations. X. J. performed STM. E. M., W. C. and X. J. wrote the manuscript with contributions from R. J. M. P. S., R. J. M. and C. M. F. were responsible for conceptualization and guidance of the research.

## Conflicts of interest

There are no conflicts to declare.

## Supplementary Material

SC-013-D1SC05127J-s001

SC-013-D1SC05127J-s002

SC-013-D1SC05127J-s003

SC-013-D1SC05127J-s004

SC-013-D1SC05127J-s005

SC-013-D1SC05127J-s006

SC-013-D1SC05127J-s007

SC-013-D1SC05127J-s008

SC-013-D1SC05127J-s009

SC-013-D1SC05127J-s010

SC-013-D1SC05127J-s011

SC-013-D1SC05127J-s012

SC-013-D1SC05127J-s013

SC-013-D1SC05127J-s014

SC-013-D1SC05127J-s015

SC-013-D1SC05127J-s016

SC-013-D1SC05127J-s017

SC-013-D1SC05127J-s018

SC-013-D1SC05127J-s019

SC-013-D1SC05127J-s020

SC-013-D1SC05127J-s021

SC-013-D1SC05127J-s022

SC-013-D1SC05127J-s023

SC-013-D1SC05127J-s024

SC-013-D1SC05127J-s025

SC-013-D1SC05127J-s026

SC-013-D1SC05127J-s027
