# Supplementary material for: Toward benchmarking theoretical computations of elementary rate constants on catalytic surfaces: formate decomposition on Au and Cu
Source: Chem Sci. 2021 Dec 21;13(3):804–15. doi: 10.1039/d1sc05127j (PMC8768843; doi:10.1039/d1sc05127j)
Supplement: SC-013-D1SC05127J-s001 [file SC-013-D1SC05127J-s001.pdf]

Electronic Supplementary Material (ESI) for Chemical Science.
This journal is © The Royal Society of Chemistry 2021

Electronic Supplementary Information

Toward benchmarking theoretical computations of elementary rate constants on catalytic surfaces: formate decomposition on Au and Cu

Eri Muramoto[a*], Wei Chen[b,c*], Xiwen Jia[c], Cynthia M. Friend[a,c], Philippe Sautet[d,e§], Robert J. Madix[a#]

[a] John A. Paulson School of Engineering and Applied Sciences, Harvard University, Cambridge, MA 02138, USA
[b] Center for Functional Nanomaterials, Brookhaven National Laboratory, Upton, NY 11973, USA
[c] Department of Chemistry and Chemical Biology, Harvard University, Cambridge, MA 02138, USA
[d] Department of Chemical and Biomolecular Engineering, University of California, Los Angeles, Los Angeles, CA 90095, USA
[e] Department of Chemistry and Biochemistry, University of California, Los Angeles, Los Angeles, CA 90095, USA

[*] Equal contribution
[§] Email: sautet@ucla.edu
[#] Email: rmadix@seas.harvard.edu

Quantitative analysis of temperature-programmed reaction spectra

A) B) C)

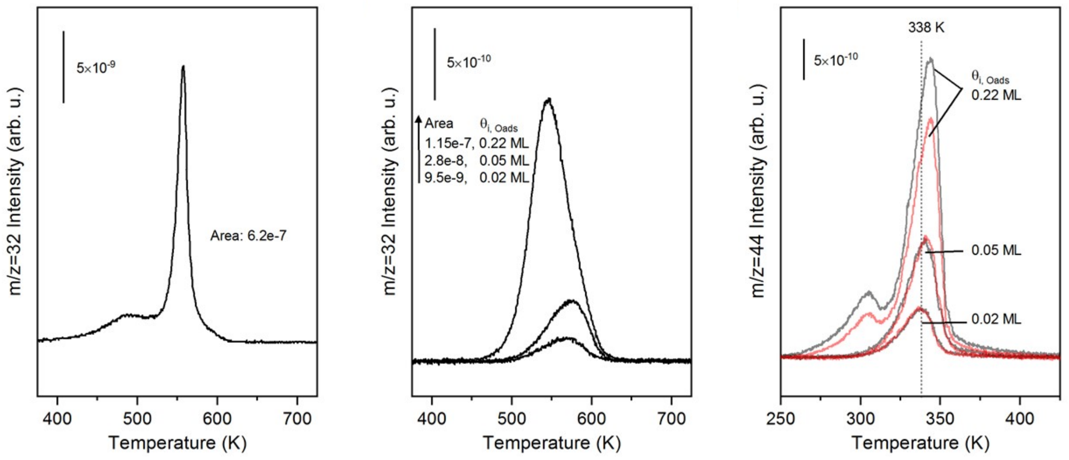

Figure S1. The coverages of atomic oxygen and formate on Au(110) were calculated by quantitative analysis of temperature-programmed reaction spectra. A) Temperature-programmed reaction spectrum obtained after exposing the clean surface to excess amount of O_3 at 300 K in order to prepare saturation coverage of atomic oxygen. At the saturation coverage a peak at 560 K with a shoulder at 480 K is obtained and the relative integrals of the primary peak and shoulder is 1:0.2 (1.2 ML).[1] B) Coverages of atomic oxygen below saturation were calculated by comparing the integrals to the integral of saturation peak in A. C) The reproducibility of formate decomposition was confirmed by performing several temperature-programmed reaction experiments at the same coverage and heating rate.

The amount of CO_2 evolution was quantified using the integral of m/z=44 peak. The integrals were used to calculate the formate coverage with an assumption that 0.05 ML of atomic oxygen produces 0.1 ML of formate and that the CO_2 desorption scales linearly with formate coverage. These coverages were also confirmed by comparing the CO_2 integrals to that of oxygen saturation peak after corrections to account for □molecular fragmentation and differences in the ionization cross-section and the mass spectrometer transmission and detection coefficients, using constants listed in Table S1.

During collection of TPD/TPRS data the sample was placed in front of the quadrupole mass spectrometer, whose ionizer and quadrupole mass filters were contained in a cylindrical shroud with an aperture at the end. The aperture (5.6 mm diameter) and sample (8 mm diameter) were concentric, and the plane of the aperture was parallel to the sample surface. The distance between them was approximately 5 mm. This setup enabled selective sampling of the species desorbing from the sample, with the mass spectrometer collecting 0-54° polar angles of desorption. If desorption from the surface was sub-cosine (angular distribution with $\cos^n\theta$, $n<1$), then the amount of product that enters the mass spectrometer would be less than for either a cosine distribution or for desorption peaked along the surface normal ($n=1$, $n>1$). If, for example, formic acid desorbed with an angular distribution with enhanced flux parallel to the surface, then the amount of formic acid detected would be reduced. This was not corrected for in this work because the angular distribution of formic acid desorption from Au(110) is not available in the

literature. However, the exact ratio of different products would not affect the conclusions of the work.

Table S1. Mass spectrometry quantitative analysis constants

m/z	Transmission Coefficient[*1]	Detection Coefficient[*2]	Molecule	Ionization Cross-section (70 eV)	Signature mass/total signal for molecule
0-20	1	1.5	O_2	2.441[*3]	0.88 (m/z=32)[*5]
21-30	1	1	CO_2	3.521[*3]	0.83 (m/z=44)[*5]
31-40	1	0.9	H_2O	2.275[*3]	0.81 (m/z=18)[*5]
41-50	1	0.8	HCOOH	5.09[*4]	0.1 (m/z=46)[*6]
			CO	2.516[*3]	0.95 (m/z=28)[*5]

[*1,2]Adapted from UTI manual
[*3]Calculated at an incident electron voltage of 70 eV from the NIST database
[*4]Adapted from Reference 2
[*5]Calculated using mass spectra from the NIST database
[*6]Measured experimentally in this work

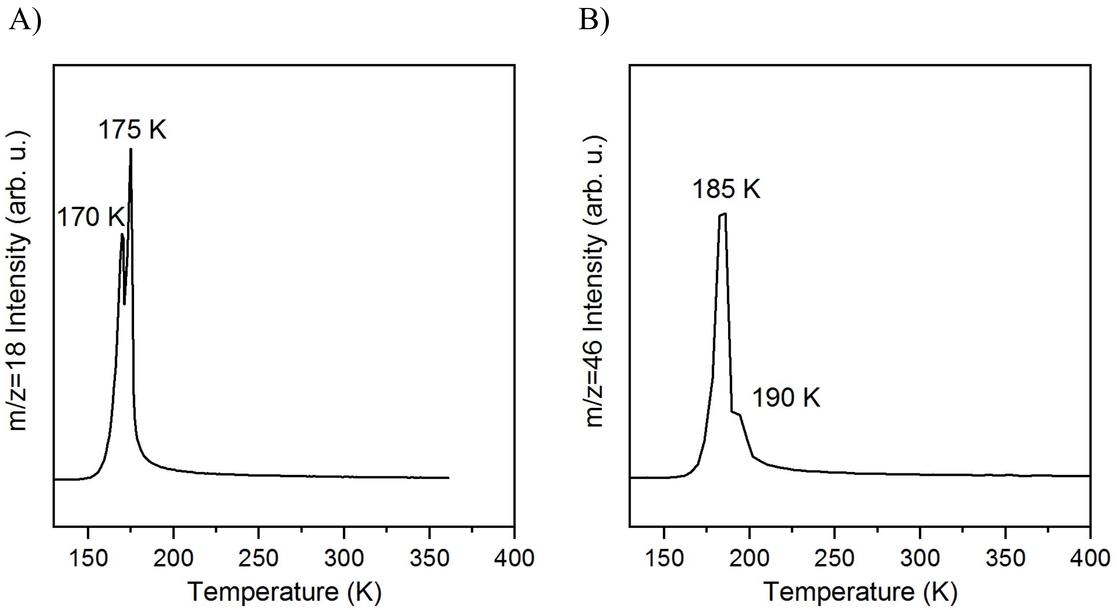

Figure S2. Temperature-programmed desorption spectra of A) water and B) formic acid on clean Au(110) showing the desorption of both species below 200 K. Lower temperature peaks are desorption from multi-layers and higher temperature peaks are desorption from the monolayer. Water was condensed at a sample temperature of 115 K and formic acid was condensed at 125 K. Heating rate was 3 K/s.

Detailed experimental methods for kinetic parameter determination

The order for the decomposition reaction of formate was determined by detailed analysis of the temperature-programmed reaction spectra.

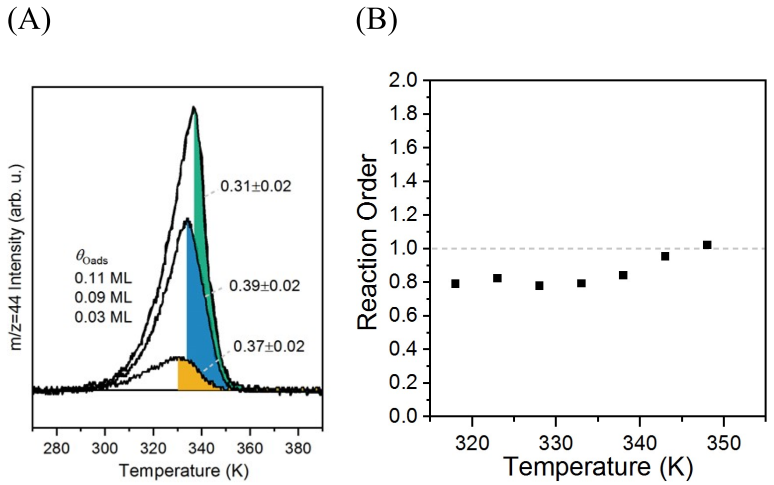

Figure S3. Formation of CO_2 from formate on Au(110) follows simple first-order kinetics at low coverages, based on analysis of the temperature-programmed reaction spectra. (A) The ratio of the integral above the peak temperature *Tp* to the total integral of the peak, $\int_{T_p}^{\infty} \frac{d\theta}{dT} dT \Big/ \int_{0}^{\infty} \frac{d\theta}{dT} dT$, is 1, 1/e≈0.37, and 0.53 for zeroth, first, and second order, respectively. A ratio of 0.37±0.02 was obtained for an initial oxygen coverage of 0.03 ML, which is in excellent agreement with the value for n=1. Ratios of 0.39±0.02 and 0.31±0.02, were obtained for initial oxygen coverages of 0.06 and 0.09 ML, respectively. The former may be higher because of the attractive interaction between formate species and the latter is likely lower because of the contribution from the second peak. (B) ln(r_d) read from spectra collected at initial oxygen coverages of 0.02, 0.03, 0.05 and 0.12 ML are plotted against ln(θ) for seven temperatures between 318 K and 348 K. Reaction order approaches 1.0 with increasing temperature and decreasing coverage.

For accurate measurements, seven heating rates ranging over two orders of magnitude, 0.1, 0.3, 0.7, 1, 2.2, 6 and 10 K/s were used (Figure S5). Activation energies were obtained from the slope of $ln(\beta/T_p^2)$ vs. $1/T_p$ by least squares fitting and pre-exponential factors were obtained by substituting the seven pairs of β and into $ln\left(\frac{\beta}{T_p{}^2}\right) = ln\left(\frac{RA}{E_a}\right) - \frac{E_a}{R}\left(\frac{1}{T_p}\right)$. The mean and standard deviation of the seven pre-exponential factors are reported in the main text.

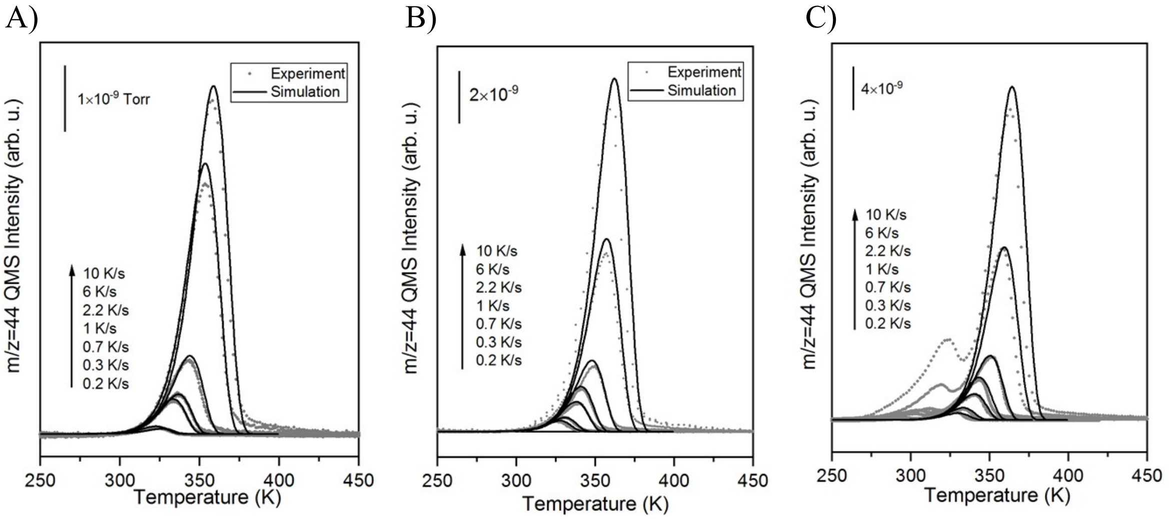

Figure S4. Temperature-programmed reaction spectra obtained in experiment and those simulated using the Polanyi-Wigner equation are in good agreement. The formate coverages are A) 0.04 ML, B) 0.10 ML and C) 0.25 ML. Seven heating rates over two orders of magnitude were used to ensure accurate measurements. The kinetic parameters obtained by the heating rate variation analysis were substituted into the equation. The integral of each experimental spectrum was used as the initial coverage. The tail on the higher temperature side is attributed to decomposition of formate that has a higher activation energy for decomposition and not due to limited pumping, as the same tail is also present in spectra collected at low heating rates.

Coverage-dependence of activation energy

To estimate the coverage dependence of the activation energy, the Redhead method and the inversion-optimization analysis were used.

At 1 K/s, peak maximum temperatures *Tp* between 331 K and 339 K give the following activation energies when pre-exponential factors of 2×10^{13} s^{-1} – 2×10^{15} s^{-1} are assumed. *Tp*, pre-exponential factor and heating rate were substituted into the Redhead equation to determine the activation energy. This shows that if the pre-exponential factor is constant or only varies within an order of magnitude around 2×10^{14} s^{-1}, the variation in the activation energy would remain within 10% of the value at 331 K.

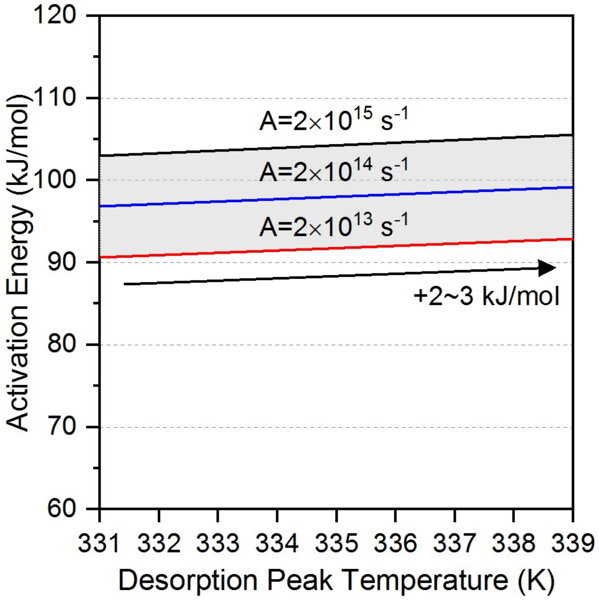

Figure S5. Activation energy determined from desorption peak temperatures by Redhead method.

The inversion-optimization method[3] can be used to extract coverage-dependent activation energy by assuming a constant pre-exponential factor from the inverted Polanyi-Wigner equation for first order processes:

$$E_a(\theta) =- RTln\left(-\frac{d\theta/dt}{A\theta}\right)$$

At 0.17 ML of formate, an activation energy of 100 kJ/mol with a variation less than 4% when coverage increases from 0 ML to 1 ML was obtained for A=2×10^{14} s^{-1} (Figure S7). This combination of activation energy and pre-exponential factor is in great agreement with what was obtained by the heating rate variation method.

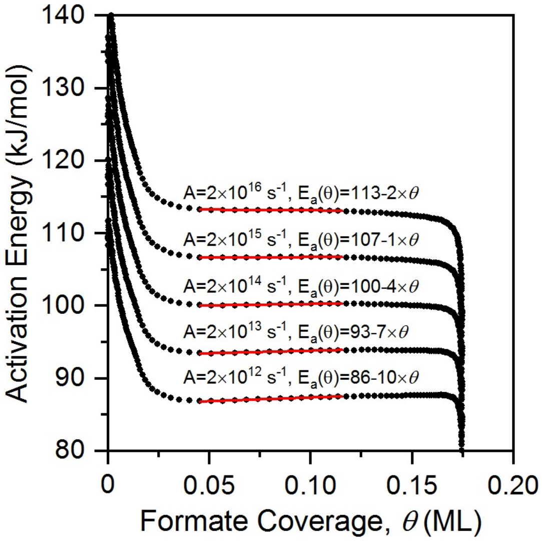

Figure S6. The temperature-programmed reaction spectrum for 0.17 ML formate was inverted using the Polanyi-Wigner equation to obtain the E(coverage) curve with five different pre-exponential factors.

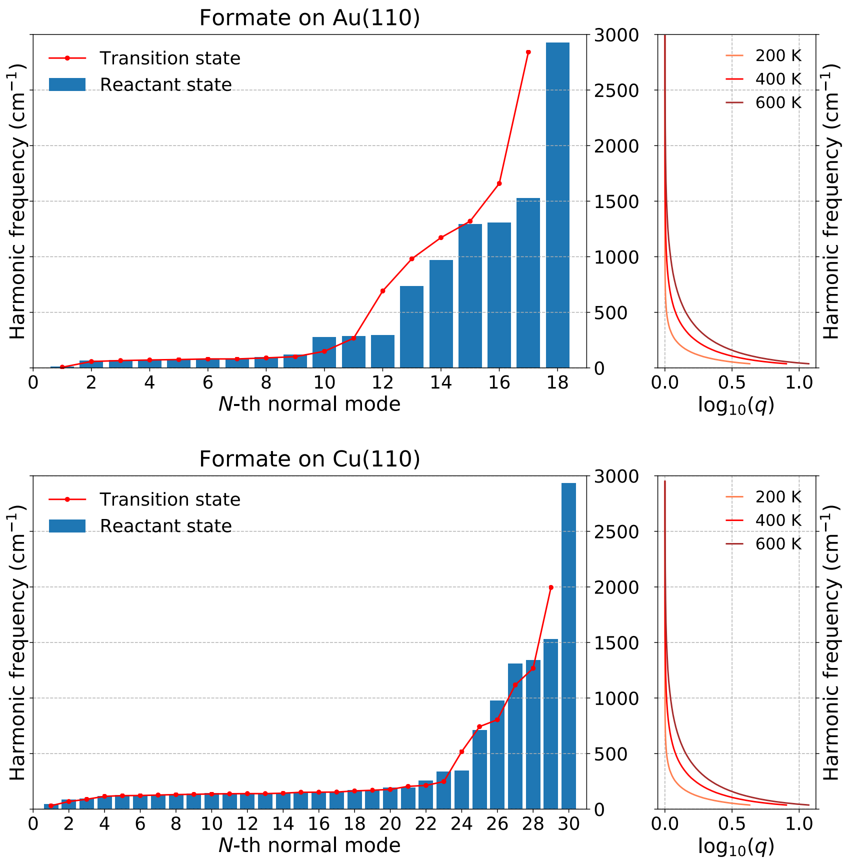

Figure S7. DFT-calculated harmonic frequencies (wavenumber, in cm^{-1}) of the normal modes of the reactant and transition states for formate decomposition on Au(110) (upper) and on Cu(110) (lower). The modes are sorted in the ascending order of the frequencies. Subfigures on the right side, with the *y*-axis aligned with the left subfigures, show the dependence of harmonic partition function (*x*-axis, in $\log_{10}$ scale) on the harmonic frequencies (*y*-axis) at $T = 200$, 400, and 600 K.

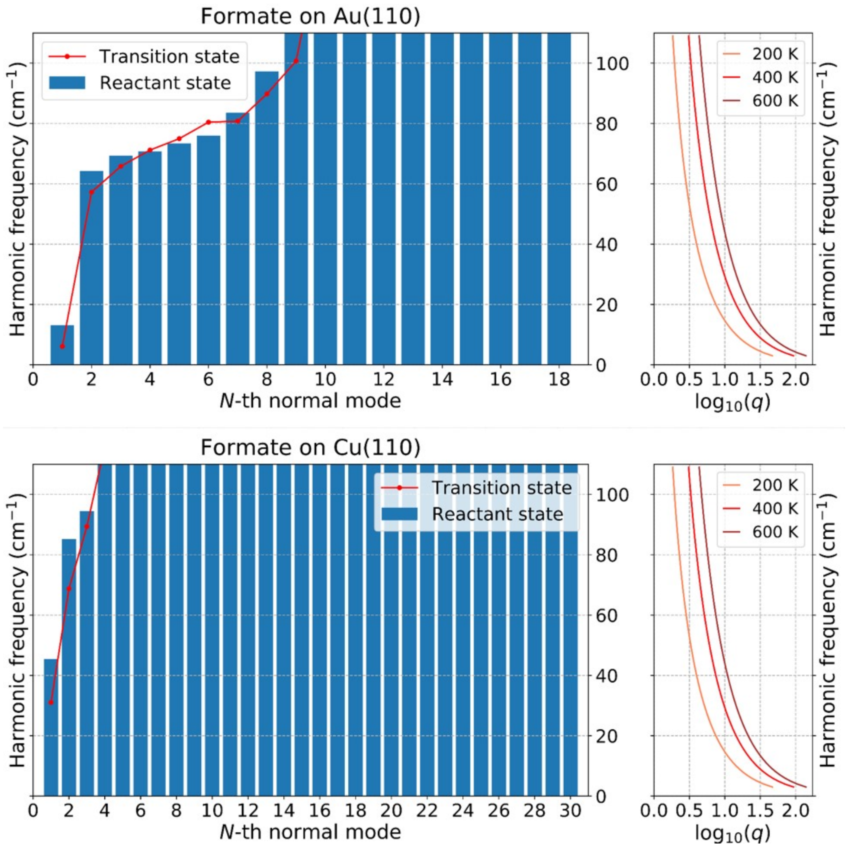

Figure S8. The same as Figure S8 except for the *y*-axis scales.

The potential errors in the predicted harmonic frequencies can be far beyond a few cm^{-1}. In this work we converged the reactant and transition state structures to a very small force threshold, in order to reach close to the limit of the accuracy. Given that on both surfaces all the three lowest frequency modes in the reactant state become softer in the transition state, we think that this trend is robust. And their errors may cancel to a great extent in calculating Q^{TS}/Q^{RS}. Another issue is that it might be problematic to treat the frustrated rotations by harmonic approximation. In our previous work[4] we clearly demonstrated the molecule size dependence of this problem. Formate is comparable in size to methoxy, where treating the frustrated rotation harmonically was sufficient.

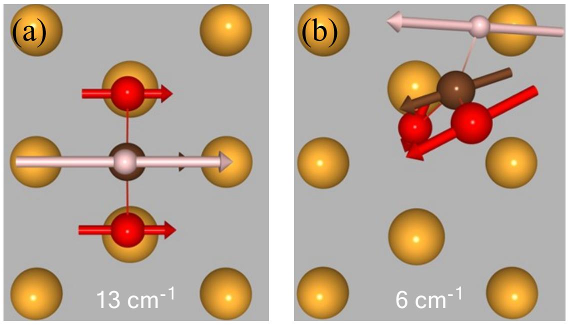

Figure S9. Top views of the displacement vectors of atoms in the lowest frequency normal modes of the (a) reactant and (b) transition states for formate decomposition on Au(110). The harmonic frequencies are listed. Each arrow indicates the direction and relative amplitude of displacement of the atom that it penetrates through.

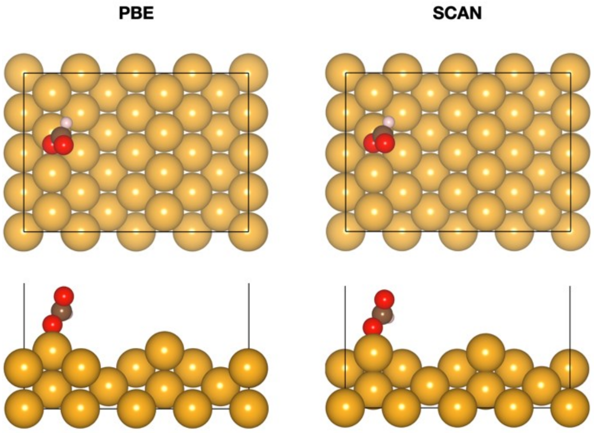

Figure S10. Comparison of transition state structures of formate on Au, calculated by (left) GGA-PBE and (right) SCAN functionals. The difference is subtle.

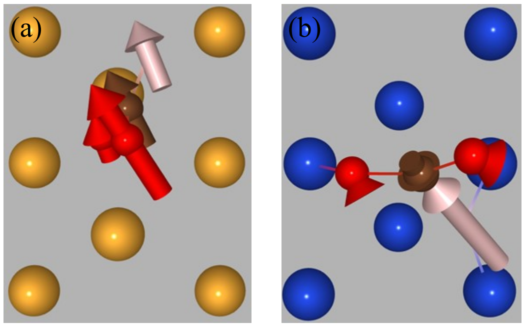

Figure S11. Top views of the displacement vectors of atoms in the transition mode of the transition state on (a) Au(110) and (b) Cu(110).

References

1 J. M. Gottfried, K. J. Schmidt, S. L. M. Schroeder and K. Christmann, *Surf. Sci.*, 2003, **525**, 184–196.

2 P. Możejko, *Eur. Phys. J. Spec. Top.*, 2007, **144**, 233–237.

3 S. L. Tait, Z. Dohnálek, C. T. Campbell and B. D. Kay, *J. Chem. Phys.*, 2005, **122**, 164707.

4 W. Chen, L. Sun, B. Kozinsky, C. M. Friend, E. Kaxiras, P. Sautet and R. J. Madix, *J. Phys. Chem. C*, 2020, **124**, 1429–1437.
